# Bootstrapping and Persuasive Argumentation

**DOI:** 10.1007/s10503-023-09627-4

**Published:** 2024-01-19

**Authors:** Guido Melchior

**Affiliations:** Department of Philosophy, University of Graz, Heinrichstrasse 26/5, 8010 Graz, Austria

**Keywords:** Bootstrapping, Argumentation, Persuasiveness, Rationality, Knowledge

## Abstract

That bootstrapping and Moorean reasoning fail to instantiate persuasive argumentation is an often informally presented but not systematically developed view. In this paper, I will argue that this unpersuasiveness is not determined by principles of justification transmission but by two straightforward principles of rationality, understood as a concept of internal coherence. First, it is rational for S to believe the conclusion of an argument because of the argument, only if S believes sufficiently many premises of the argument. Second, if S doubts that a source *O* is reliable and believes that information *i* is delivered by *O*, then S rationally suspends judgment about the truth of *i*. This paper aims to accomplish two tasks. First, it provides a thorough analysis of why bootstrapping argumentation is not an instance of rational persuasion. Second, it contains a more general theory about preconditions and limits of persuasive argumentation.

## Introduction

Bootstrapping, often also labelled as easy knowledge or epistemic circularity, is a much-discussed process in contemporary epistemology.^1^ It is an intuitively flawed method of reasoning, but widely accepted epistemic principles seem to support its legitimacy, namely that there is fundamental knowledge or justification and that it can be extended via induction. There are different explanations on the market of why bootstrapping is flawed or seems flawed. One explanation focuses on the dialectical defectiveness of bootstrapping. In this paper, I will take a fresh perspective on bootstrapping by analyzing it within the context of argumentation between two interlocutors. I will argue that the persuasive defectiveness of bootstrapping relies on two internal rationality principles that jointly entail that it is irrational to being persuaded via bootstrapping arguments. These results have a significant impact on argumentation theories, since they offer an explanation of why certain epistemically circular argumentations are not persuasive and a resulting account about the limits of persuasive argumentation.

Bootstrapping was originally introduced as a process of reasoning rather than a process of argumentation. Vogel (2000 and 2008) presents the case of Roxanne who comes to believe that her gas gauge is reliable by repeatedly looking at it and inferring as follows:

**Gas gauge**

(1) Tank is full at t_1_ (Reading the gauge)

(2) Gauge reads ‘F’ at t_1_ (Perception)

(3) Gauge reads ‘F’ at t_1_ & Tank is full at t_1_ (Logical inference)

(4) Gauge reads accurately at t_1_ (Logical inference)

(5) Repeat […]

(6) Gauge is reliable (Induction)

Vogel argues that each step of Roxanne’s bootstrapping process results from a reliable process and concludes that process reliabilists are committed to accepting the implausible consequence that bootstrapping can yield justification and/or knowledge. Reading the gas gauge is the process leading to belief (1) that the tank is full at t_1_, perception is the process leading to belief (2) that the gauge reads ‘F’ at t_1_, and logical inference is the process leading to the belief that the gauge reads accurately at t_1_. Roxanne’s reasoning process is an instance of the more general bootstrapping process where a subject reasons that a source *O* is reliable by repeatedly consulting *O* and drawing inferences.

**Bootstrapping**

P_1_: *p*_1_ (delivered by *O*)

P_2_: *O* indicates that *p*_1_

P_3_: *p*_1_ ∧ (*O* indicates that *p*_1_)

Repeat for *p*_2_…*p*_n_

C:* O* is a reliable source.

I understand bootstrapping as a process of inductive or deductive reasoning from information delivered by a source to the conclusion that this source is reliable.^2^ Importantly, bootstrapping can also be used for inferences to the conclusions that one’s own sense apparatus is reliable and that one is not in a skeptical scenario. This version of bootstrapping is an instance of Mooreanism which I will call here *Moorean bootstrapping*.^3^ It is essential for bootstrapping reasoning that, for settling the question whether a source *O* is reliable, information from *O* is used. Hence, bootstrapping processes are distinct from other intuitively correct process of determining whether a source is reliable that involve information from another source, for example when the reliability of a thermometer is checked by using another thermometer that is known to be reliable.

Bootstrapping is usually discussed as a process of *reasoning* about the reliability of a source. Nevertheless, it has been occasionally noted that bootstrapping arguments are dialectically ineffective.^4^ However, dialectical ineffectiveness is primarily a feature of interpersonal interactions such as argumentation rather than one of mere reasoning. In contrast to approaches that center on the unpersuasiveness of bootstrapping reasoning, this paper will primarily focus on explaining the unpersuasiveness of bootstrapping *argumentation*. I argue that the unpersuasiveness of bootstrapping argumentation is based on two rationality requirements that people usually follow. Thus, people are not only usually unpersuaded by bootstrapping arguments, they *rationally* remain unpersuaded.

## Persuasion and Argumentation

In this paper, I focus on persuasion via* argumentation*. Persuasion however, is a more general phenomenon and not all instances of persuasion involve argumentation. We can persuade others, for example, simply because they trust us. So before analyzing argumentation, let me provide a more detailed account of persuasion.^5^ I understand persuasion as an interpersonal act where a persuading subject, to whom I refer as A, makes another subject, to whom I refer as B, believe a proposition. When I talk about persuasion, I focus on processes of belief-formation rather than on processes of knowledge acquisition or warrant-transmission. Let me provide the following definition of persuasion:

**Definition: Persuasion**

A persuades B that *p* via verbal action *c* iff A performs *c* and B believes that *p* because of *c*.^6^

This definition is meant to capture the idea that B believes that *p* because of the content of A’s utterances and to exclude cases of deviant causation e.g., where B believes that *p* simply because of the sound of the argument presented. Adding an additional requirement that B believes that *p* because she correctly understood the content of *c* could explicitly exclude such cases of deviant causation. Moreover, further refinements might be required for excluding cases of deviant causation.

We can ascribe the capacity of persuasion or the status of being persuaded not only to natural persons but also to groups or institutions. For the sake of simplicity, I will focus here on natural persons. In the philosophically interesting case of Moorean bootstrapping only one person is involved. I will investigate Moorean bootstrapping later as a process that mimics ordinary argumentation involving two persons in that an inner believer aims at persuading an inner skeptic via a Moorean argumentation that the skeptical hypothesis is false.

I am primarily interested in forms of persuasion via argumentation.^7^ However, for the sake of terminological unity, I use the notion of persuasion in a rather wide sense, which does not match our natural language understanding in all cases. Mere utterances, which do not involve any argumentation, can also count as instances of persuasion according to my account.^8^

Since persuasion is a belief forming process, let me provide some clarificatory remarks on doxastic attitudes. We can distinguish three different doxastic attitudes towards *p*: believing that *p*, rejecting that *p* (which I assume to be equivalent to believing that ~ *p*)^9^ and suspending judgment about *p*. When S suspends judgment concerning *p*, S entertains *p* and considers whether *p* is true but refrains from believing and from rejecting it. We typically suspend judgment concerning *p* when we think that we have equally good evidence in favour of and against *p*, a simple case being if we think that we do not have any evidence whatsoever concerning *p*. As for each of these three doxastic attitudes, S entertains the target proposition (or would at least entertain the proposition when being confronted with it.) We must distinguish these three attitudes from *being unaware* of the proposition that *p* which is a lack of any doxastic attitude towards *p*. In particular, we have to distinguish cases of being unaware that *p* from suspending judgment that *p*. In contrast to believing, rejecting, or suspending judgment, if S is unaware of *p*, then S has not entertained *p* and might not even be cognitively able to grasp the content of *p*.

Implicitly believing that *p* is often understood as the disposition to explicitly believe that *p* when this proposition is entertained or the disposition to affirm that *p* when one is asked whether *p* is true. Accordingly, one might suggest that cases of being unaware that *p* are cases of having an implicit doxastic attitude towards *p*. However, S might simply fail to grasp the content of *p*, and, in this case, S does not affirm, reject, or actively suspend judgment about *p* when being confronted with *p*. For the purposes of this paper, it is not necessary to draw a sharp distinction between being unaware and having implicit doxastic attitudes. In both cases, bootstrapping argumentation is not a persuasive mechanism, though for different reasons, as we will see. For the sake of terminological simplicity, let me introduce doubting as a technical term as follows:

**Definition: Doubting**

S doubts that *p* if S rejects that *p* or suspends judgment about whether *p* is true.

If S doubts that *p*, then, by definition, S holds a doxastic attitude towards *p*, either a neutral attitude when suspending judgement or a negative attitude in the case of rejection. Lack of awareness, which does not involve any doxastic attitude towards *p*, is, by definition, not an instance of doubting.^10^ This definition of doubting is presumably not entirely in line with our natural language understanding, according to which doubting means suspending judgment about a proposition rather than rejecting it.

Given the three doxastic attitudes plus unawareness, we can distinguish three different types of persuasion via verbal action *c*.

(1) B initially *rejects* that *p* and A performs action *c* and B believes that *p* because of *c*.

(2) B initially *suspends judgment* concerning *p* and A performs action *c* and B believes that *p* because of *c*.

(3) A performs action *c* and B believes that *p* because of *c* and B was *not aware* that *p* prior to being confronted with *c*.

In case (1), A transforms B’s negative doxastic attitude towards *p* to a positive one and, in case (2), B’s neutral doxastic attitude. In case (3), A causes a doxastic attitude in B towards *p* where B previously held no doxastic attitude towards *p*. Given the definition of unawareness provided, this case might involve A making B grasp the content of *p*.^11^

Which type of persuasion occurs between A and B often depends on the conversational context. When A simply *tells* B that *p*, B might not have been aware of *p* prior to being presented with *p*. However, when A and B argue about *p*, then *p* has been introduced prior to engaging in the act of argumentation and, thus A and B are already aware of *p*. In this latter case, persuasion is typically of type (1) or (2).

Disagreement between two persons requires different doxastic stances towards the same proposition *p*, e.g. A believes that *p* and B rejects that *p* or A believes that *p* and B suspends judgment about whether *p* is true.^12^ Moreover, we can note that persuasion is different from meeting or defeating someone’s objections. A can successfully meet or defeat B’s objection against *p* according to objective standards while still failing to make B believe that *p*. In this respect, a theory of persuasion must be distinguished from theories of defeasibility.^13^

Let us have a closer look at some paradigmatic instances of persuasion. Persuasion can happen in different ways, and argumentation is a paradigmatic form of persuasion. The simplest acts of persuasion that *p* are mere utterances that *p*. These instances of persuasion involve trust. Accordingly, I will define them as follows:

**Definition: Persuasion via Trust**

A persuades B via trust that *p* iff A utters that *p* and B believes that *p* because of A’s utterance that *p*.^14^

B can trust A concerning *p* for various reasons. First, B can trust A automatically, e.g., as a matter of behaviour. For example, a child might automatically believe what her parents tell her, exhibiting her trust in them.^15^ Moreover, B might trust A concerning *p* because she trusts A concerning the whole domain of *p*. B might believe that A is competent concerning this domain, e.g., when we believe utterances of experts in their area of competence even if they do not provide further argumentation or proof. This kind of trust-based persuasion merely by uttering a proposition is the simplest case.

However, in many cases A cannot persuade B that *p* merely by uttering *p*. In these cases, an argument, demonstration, or proof for the truth of *p* is required. So let me come to the central notion of persuasion via argumentation. I define *argumentations* as at least partly verbal acts that involve premises that are uttered (or implied) in support of a conclusion *p*.^16^ These acts might be purely verbal or they might also involve non-verbal demonstrations.^17^ Since bootstrapping involves inferences, cases of persuasion that involve argumentation are more important here than persuasion via mere demonstration. Let me define persuasion via argumentation as follows.

**Definition: Persuasion via Argumentation**

A persuades B via argumentation that *p* iff A presents an argumentation for *p* and B believes that *p* because of this argumentation.

Let me provide some examples. If A persuades B by presenting an abductive argument that it has been raining by uttering, “The street is wet, and so it must have been raining,” then A persuades B via *argumentation*. This is also the case if A persuades B that it has been raining by demonstrating that the street is wet (e.g., by pointing at it) and uttering, “The street is wet, and so it must have been raining.” However, if A persuades B that the street is wet merely by pointing towards the wet street and uttering that the street is wet, then A persuades B via *demonstration* that the street is wet.^18^

I propose here a rather narrow conception of persuasion via argumentation, since argumentation and persuasion via argumentation usually involve the exchange of various arguments, supporting arguments, and counter-arguments. However, I argue that bootstrapping argumentation, by its very structure, violates criteria of internal rationality, and is, therefore, rationally unpersuasive. Thus, no supporting argument can compensate for this deficit and no counter arguments against the premises of the bootstrapping argument need to be presented in order to justify doubting the conclusion. Consequently, more complex forms of argumentation need not to be considered for making the point concerning the unpersuasiveness of bootstrapping. In this respect, the narrow definition of persuasion via argumentation is sufficient for the purposes of this paper.

Persuasion via argumentation requires that B believes that *p because of* the argumentation provided by A. A does not persuade B via argumentation if A presents an argument for *p* and B believes that *p* for other reasons, e.g., because of A’s voice. These are still instances of persuasion, but not of persuasion via argumentation. In a wider sense, a presented argument can also cause a belief that *p* if B believes that *p* because of the sound of the argument or because of some other superficial features. In this wider sense, A can persuade B that *p* via argumentation even if B does not understand the argument or does not even recognize it as such. Hereinafter, I will understand persuasion via argumentation in a narrower sense that requires that B recognizes that A presents an argument, understands A’s argument, and believes that *p* because of the understood content of the argument.^19^

I argue in this paper that bootstrapping argumentation violates criteria of internal rationality and, therefore, does not qualify as persuasive argumentation. In this paper, I understand rationality requirements as requirements concerning what one ought to believe given the beliefs that one already has. For example, given that S believes that *p* and believes that *p* entails *q* it is rational for S to believe that *q*. Furthermore, given that S believes that *p* and believes that *q* is incoherent with *p*, it is rational for S to reject that *q*.^20^ These rationality requirements are merely criteria of internal coherence and not criteria of justification. For example, it is rational for S to believe that *q* given that S believes that *p* and that *p* entails *q*, regardless of whether S’s beliefs that *p* and that *p* entails *q* are justified or not.^21^ Such rationality requirements can also be formulated for interlocutors of argumentations. Here is a first crucial rationality requirement RP concerning argumentation:

**RP**

B rationally believes that *c* because of A’s argumentation consisting of premises *p*_1_…*p*_n_ and conclusion *c*, only if B believes premises *p*_1_…*p*_n_.^22^

Notably, RP can be realized in different ways. A standard way of persuading someone in line with RP is that B believes the premises *p*_1_…*p*_n_ of A’s argumentation prior to being confronted with A’s argumentation and A persuades B mainly by establishing a connection between *p*_1_…*p*_n_ and *c* that B did not recognize. However, B might also trust A concerning the premises such that A makes B believe premises *p*_1_…*p*_n_ by uttering them. In actual argumentation, a combination of these mechanisms is often at play. RP is meant to be compatible with all these cases, and not only with B believing the premises prior to being confronted with A’s argumentation. Thus, RP is properly understood as stating that B rationally believes that *c* because of A’s argumentation only if B believes the premises of A’s argumentation at least after being confronted with A’s argumentation.

One might argue that not all kinds of argumentation are such that they require belief in the premises for being persuasive. For example, when being confronted with a reductio argument, we only need to take the premises hypothetically into account. However, I assume that reductio argumentations are actually embedded in more extensive argumentations for which RP holds. In the case of reductio argumentations, it is (often implicitly) assumed that *p* is false if *p* entails an absurd consequence. S hypothetically supposes that *p* in order to show that *p* entails *q* which is argued to be absurd, and from this it is concluded that *p* is false. However, in these cases of reductio argumentation, *p* need not actually be accepted. Rather, *p* is hypothetically presupposed to be the case, with the primary argument offered for the entailment relation between *p* and *q*.

RP states that it is irrational to believe the conclusion of someone’s argumentation because of the argumentation without believing the premises, for example, if we fall prey to wishful thinking about *c*. In this case, we might believe that *c* because of an argumentation even if we do not believe the premises. I assume that we not only rationally but also *usually* form beliefs in line with RP. Admittedly, this is an empirical hypothesis. We are not perfectly rational when engaging in argumentation and consequently violate RP in various ways. Nevertheless, I think that we can correctly say that we usually, though not always, follow RP.^23^

## Bootstrapping as Unpersuasive Argumentation

Based on the preliminary remarks on persuasion and argumentation, we will, in this section, reveal the reasons for the persuasive defectiveness of bootstrapping argumentations. A presents a bootstrapping argumentation to B if A argues for the reliability of a source *O* by using information delivered by *O* as premises of the argumentation. A might only refer to the premises delivered by *O*, e.g., by uttering that the gas gauge makes the particular indications, or A might also demonstrate to B that* O* makes these indications, e.g., by pointing to the gauge’s indications. By definition, persuasion via bootstrapping requires that B believes that *O* is reliable because of the bootstrapping argumentation. Given different doxastic attitudes of B as potential starting points, three cases of persuasion concerning *O*’s reliability can be distinguished: transition from rejecting that* O* is reliable to believing it, transition from judgement suspension to believing it, and transition from unawareness to believing it.

With RP, I have already introduced a general rationality requirement for argumentation. Let me now introduce a second rationality requirement, RR, which concerns the reliability of sources:

**RR**

If S doubts that *O* is reliable and believes that information *i* is delivered by *O*, then it is rational for S to suspend judgment about the truth of *i* based on O delivering that *i*.^24^

RR is a rationality requirement that concerns the internal coherence of one’s belief system. Let me first provide some clarificatory remarks before explicating the significance of this principle for argumentation: First, for rationally suspending judgment about *i*, it is crucial that we *believe* that *i* is delivered by *O*, not that *O* actually delivers that *i*. We might falsely believe that *i* is delivered by *O*, and yet we rationally suspend judgment about whether *i* is true. Moreover, if *i is* delivered by *O* but we do not believe that either because we reject that *i* is delivered by *O* or because we are unaware of it, then we might rationally believe that *i* is based on *O* although we do not believe that *O* is reliable.^25^ Second, whether we rationally suspend judgment about the truth of *i* does not depend on the actual unreliability of *O* but on whether we *doubt* that *O* is reliable. Even if *O* is reliable, we rationally suspend judgment about the truth of *i* if we mistakenly doubt the reliability of *O*. Third, if one merely rejects that *O* is reliable and believes that information *i* is delivered by *O*, then one rationally *suspends judgment* about the truth of *i*. One does not rationally believe that *i* is false. This belief in the falsity of *i* is rational only if S believes the stronger proposition that* O* always (or in most cases) delivers false information. Fourth, RR is restricted to suspending judgment about or rejecting the reliability of *O*. It does not cover unawareness about the reliability of *O*. We will see soon that RR does not hold for this case. Fifth, RR is only a claim about the rationality of doubting on the basis of a particular source. It might not be rational for S to doubt that *i* if S has information from other sources about the truth of *i*. As in case of principle RP, I assume that subjects usually though not always follow RR.

RR can also be formulated in terms of defeaters. Defeaters remove one’s justification for *p*.^26^ Pollock (1986) distinguishes between rebutting and undercutting defeaters. Suppose that S has justification to believe that *p* based on evidence *e*. A rebutting defeater for *p* removes S’s justification for *p* directly by presenting evidence for ~ *p*. An undercutting defeater does so by stating that *e* does not provide evidence for *p*. In contrast to rebutting defeaters, undercutting defeaters do not provide evidence for ~ *p*. RR does not make any claims about justification, but about rationality understood as a phenomenon of internal coherence. In terms of defeaters, RR states that an undercutting mental state defeater makes it rational for S to suspend judgment about *p* on the basis of *e*.^27^

Principle RR is formulated as a general principle of rationality. However, its relevance for argumentation is obvious. If A presents an argument to B by relying on information that B believes is delivered by a source whose reliability B doubts, then it is rational for B to suspend judgment about whether this information that A uses is true. In order to show that bootstrapping is not a persuasive way of arguing, we have to show that bootstrapping argumentation does not rationally lead to a transition from rejecting, suspending judgment, or being unaware to believing. I will first handle rejection and judgment suspension, i.e., doubt, about the reliability of a source, and then reflect on unawareness. We have revealed two principles concerning our rational belief forming behaviour, RP and RR. These two principles determine that a subject is rationally not persuaded via bootstrapping argumentation that a source *O* is reliable if she doubts that* O* is reliable. Here is the explanatory argument in detail:

**Argument for the unpersuasiveness of bootstrapping argumentation**

(1) Suppose that B doubts that* O* is reliable and A presents a bootstrapping argumentation to B that *O* is reliable.

(2) If B doubts that *O* is reliable and believes that information *i* is delivered by *O*, then B rationally (and usually) suspends judgment about the truth of *i*. (RR)

(3) In cases of bootstrapping argumentation, the premises of A’s argumentation that* O* is reliable are based on information delivered by *O*. (Definition of bootstrapping argumentation)

(4) B rationally (and usually) believes that *c* because of A’s argumentation consisting of premises *p*_1_…*p*_n_ and conclusion *c*, only if B believes premises *p*_1_…*p*_n_. (RP)

(5) Therefore, B is rationally (and usually) not persuaded that *O* is reliable via bootstrapping.

Let’s have a look at a concrete bootstrapping case of the gas gauge. Suppose B doubts that gas gauge G is reliable and A aims to persuade B that G is reliable via bootstrapping argumentation. Let us see how bootstrapping is properly formulated as a process of argumentation. In the original bootstrapping case, Vogel (2000) considers processes of reasoning rather than processes of argumentation between two interlocutors. In order to understand bootstrapping *argumentation*, this process must be adopted for argumentation.

The criteria of rationality formulated here that a bootstrapping subject violates are purely subjective. Therefore, it is crucial that the bootstrapping subject also recognizes the bootstrapping process as such. This is true for paradigmatic cases of bootstrapping *reasoning*, such as Vogel’s gas gauge case, or Moorean reasoning, much discussed in the philosophical literature. They are obviously instances of bootstrapping. However, the situation is different for bootstrapping argumentation. Different formulations of bootstrapping argumentation are possible but not every form of bootstrapping argumentation makes the bootstrapping nature of the argumentation transparent to the other interlocutor. First, one might understand A’s bootstrapping argumentation as the following series of statements, which is strongly analogous to the process of bootstrapping reasoning:

**1. Opaque bootstrapping argumentation**

(1) ‘G indicates that F at t_1_.’

(2) ‘The tank is full at t_1_.’

(3) ‘Therefore, the tank reads accurately at t_1_.’

(4) …

In the case of bootstrapping as a belief forming process, S’s belief that the tank is full at t_1_ *is based* on her observation of the gas gauge reading ‘F’ at t_1_. However, this fact is not expressed in the formulation of the argumentation above. A’s argumentation does not make explicit that claim (2) that the tank is full at t_1_ is based on A’s claim (1). Thus, the bootstrapping process is not made transparent to B. A’s utterance of (2) is compatible with A having alternative evidence that the tank is full at t_1_, e.g., based on using a dipstick for measurement. Thus, B does not violate any purely internal criterion of rationality when believing that *O* is reliable because of A’s bootstrapping argumentation. Accordingly, B has various positive rational options concerning (2). B might trust A and simply believe that A has additional evidence for (2), or B might ask A what evidence A has for (2).^28^

In contrast, the bootstrapping nature of A’s argumentation is made obvious to B by the following utterances:

**2. Transparent bootstrapping argumentation**

(1) ‘G indicates that F at t_1_.’

(2) ‘Therefore, the tank is full at t_1_.’

(3) ‘Therefore, the tank reads accurately at t_1_.’

(4) …^29^

In this case, A indicates that her claim (2) is based on her claim (1) by using ‘therefore’, in contrast to the case of opaque bootstrapping argumentation.

Because of A’s indication that her claim (2) is based on (1), the bootstrapping nature of A’s argumentation is transparent to B. Given that B doubts that G is a reliable source, B will doubt that (2) on the basis of (1), and A’s bootstrapping argumentation is not rationally persuasive. B might believe that G delivers certain indications based on A’s utterance (or A’s demonstration), but due to principle RR, B does not rationally believe that these indications are true and, consequently, fails to believe that G’s indications are accurate and that G is reliable. In case 1 of opaque bootstrapping argumentation, the bootstrapping nature of the argumentation is not transparent to B and, therefore, B does not violate any internal criteria of rationality when believing that the target source is reliable based on bootstrapping argumentation. In contrast, the bootstrapping nature is transparent in case 2, and, therefore, B is irrational when believing based on bootstrapping argumentation. Thus, pattern 2 is the more appropriate understanding of bootstrapping argumentation. Similar issues do not arise for bootstrapping reasoning since in the paradigmatic cases discussed in the philosophical literature the bootstrapping nature of the argumentation is assumed to be transparent to the reasoning subject and to the judging reader. As we can see, there are subtle differences between bootstrapping as a belief-forming process and bootstrapping as argumentation that have to be taken into account.

Let me next come to the case of Moorean bootstrapping. Moorean *reasoning* is a process of forming a belief (or of acquiring justification or knowledge) about the reliability of one’s own sense apparatus via information delivered by one’s own sense apparatus. In this respect, Moorean reasoning is not a potential interaction between two actual persons as argumentations and demonstrations are. Thus, in order to understand why Moorean bootstrapping is not a persuasive way of *arguing*, we have to reconstruct it as a form of an inner argumentation between an inner believer and an inner skeptic that mimics actual argumentation between two persons as follows:^30^

**Moorean bootstrapping argumentation**

Suppose, S’s inner skeptic B doubts that S’s sense apparatus is reliable and A, S’s inner believer, aims to persuade B about its reliability via Moorean bootstrapping.

(1) A utters or demonstrates that S’s experience delivers that *p* by pointing towards S’s experience

(2) B thereby believes that S’s sense apparatus delivers that *p*

(3) A utters that, therefore, *p*

(4) B suspends judgment about whether *p* is true based on S’s experience as of *p*

(5) Therefore, B does not believe that S’s experience accurately delivers that *p*

(6) …

(7) Therefore, B does not believe via Moorean bootstrapping argumentation that S’s sense apparatus is reliable.

S’s inner skeptic cannot be rationally persuaded via Moorean bootstrapping about the reliability of S’s sense apparatus.

Bootstrapping was introduced in the philosophical literature as part of a reductio argument showing that externalist accounts of knowledge and/or justification are committed to accepting that bootstrapping can yield knowledge and/or justification. The more obvious the bootstrapping nature of a process is, the stronger the intuitions are about its defectiveness, and also about its dialectical defectiveness. In order to make the strongest possible point, the examples in the literature are chosen in a way that the bootstrapping nature of the process is maximally obvious, as in Vogel’s gas gauge case. Thus, one might object that obvious bootstrapping argumentation is rather a hypothetical argumentation scheme than one that is actually performed, and that the overall idea of bootstrapping argumentation is undermotivated. First, this take on actual bootstrapping argumentation is not in conflict with one of my general claims. This paper aims at analyzing the existing intuition that bootstrapping is epistemically defective in the sense that it is dialectically defective. This goal can be achieved without referring to actual instances of bootstrapping argumentation. Second, let me note that there are cases of real-life disagreement, for example as presented in the discussion of deep disagreement, that involve bootstrapping argumentations, albeit often in a masked way, even if the argumentations of the interlocutors are interpreted in the most charitable way.^31^

So far, we have seen why someone cannot rationally persuade a person about the reliability of a source via bootstrapping argumentation who *doubts* that the target source is reliable, i.e., who rejects that it is reliable or actively suspends judgment about its reliability. However, we have seen that we can distinguish believing that *p* not only from the two non-positive doxastic attitudes that constitute doubting but also from being unaware of *p*, which means lack of any doxastic attitude concerning *p*. If we talk about unawareness concerning a proposition, then we also have to consider unawareness about sources.

We not only believe information delivered by *O* if we believe that *O* is reliable. Often, we believe it without ever having considered whether *O* is reliable or without being in a position to do so. One might object that we implicitly believe that *O* is reliable in these cases and that we would confirm that *O* is reliable if we were confronted with the proposition that *O* is reliable. However, there are cases of believing via a source where it is dubitable that any implicit belief in the reliability of a source is involved. For example, children believe information via a source, e.g., their sense apparatus, although they do not have any concepts of sources, sense apparati, and reliability. In this case, it is implausible to claim that the child implicitly believes that the source is reliable.^32^ Thus, there are plausibly cases where a subject believes information delivered by* O* while being unaware about* O*.^33^ I understand rationality as a purely internal requirement for what one ought to believe given the beliefs that one already holds. If S does not hold any kind of doxastic attitude about a source, then there are no doxastic attitudes that can pose a restriction on whether S should or should not believe information from this source. A child who believes information via a source, e.g., via her sense apparatus, without being aware of the existence of a sense apparatus and without possessing the relevant concepts does not violate any criteria of internal coherence or rationality. Hence, the following analogy to RR for unawareness does not hold:

**The false claim: RR for unawareness**

If S is unaware that *O* is a reliable source, then rationally S does not believe information delivered by *O*.

Consequently, the following general claim about *not believing* that *O* is reliable, which also includes unawareness, also turns out to be false:

If S does not believe that *O* is a reliable source, then rationally S does not believe information delivered by *O*.^34^

Thus, subjects can rationally believe information delivered by *O* without having a belief that *O* is reliable. Does this possibility have any impact on the potential persuasiveness of bootstrapping argumentation? Can A first persuade B that *i* on the basis of *O* where B is unaware that *i* is delivered by* O* and then rationally make B believe via bootstrapping argumentation involving *i* that *O* is reliable? Arguably, A cannot. Take the case of A who persuades B that *i* on the basis of *O* but B is unaware of *O* or of the fact that *i* is delivered by *O*. I do not provide a clear definition of unawareness, so let us consider two cases separately, first, that B is unaware in that she cannot possess the required concepts of *O* and, second, that she is unaware in that she has just not paid the required attention to the fact that *i* is based on *O*. Suppose first that B does not have the capacity to form beliefs about *O*. In this case, B will trivially not believe that *O* is reliable via A’s bootstrapping argumentation. Suppose now that B believes that *i* on the basis of A’s argumentation and has the capacity to form beliefs about *O* but is unaware of *O* or of the fact that *O* delivers that *i*. If A transparently presents the bootstrapping argumentation to B following bootstrapping argumentation pattern 2, then A indicates that *i* is delivered by *O*, and because of this argumentation, B will become aware of *O*. By becoming aware of *O*, B can take different doxastic stance towards *O* being reliable. If B believes that *O* is reliable by simply becoming aware of *O* or simply because of A’s mere utterance of the conclusion that *O* is reliable, then B is trivially not persuaded that *O* is reliable via* bootstrapping argumentation*. If B doubts that *O* is reliable by becoming aware of *O*, i.e., if B rejects that *O* is reliable or suspends judgment about the reliability of *O*, by becoming aware of *O* then rationally B will retroactively put her belief that *i* into doubt. Since *i* on the basis of O is a crucial premise of A’s bootstrapping argumentation, B will rationally not believe that *O* is reliable via bootstrapping argumentation. Hence, if B believes that *i* and is unaware about *O*, B will not believe via A’s bootstrapping argumentation that *O* is reliable.^35^

To sum up: If B doubts that *O* is reliable then A cannot rationally persuade B via bootstrapping argumentation about the reliability of *O*. A also cannot rationally persuade B if B is initially unaware of *O* or of the fact that *i* is delivered by *O*. Hence, we cannot rationally persuade someone via bootstrapping argumentation that a source is reliable.

Notably, bootstrapping argumentation fails to be persuasive due to principles of rationality understood as principles about what a subject should rationally believe given the beliefs that she already holds. Usually, the persuasiveness of an argumentation crucially depends on whether the beliefs of A and B about the truth of the premises and/or the validity of the arguments used sufficiently match. If not, then persuasion via argumentation fails. In the specific case of bootstrapping argumentation, however, the argumentation fails to be persuasive due to criteria of internal rationality for B. A’s attitudes or a mismatch between A’s and B’s beliefs about the truth of premises are not crucial for the unpersuasiveness of bootstrapping argumentation. In this sense, it is a special case of unpersuasive argumentation.

Nevertheless, these cases are significant for real life. Take, for example, the case of disagreement about the age of Earth between a scientist and a creationist, which is regarded as a paradigmatic case of deep disagreement. Such cases of deep disagreement often also involve disagreement about the reliability of sources.^36^ This case is typically set up in a way that the scientist believes in the reliability of scientific evidence whereas the creationist believes that the bible as a source of information potentially overrides all other sources. Accordingly, in this case persuasive argumentation also involves argumentation about the reliability of the sources used. Since there is no further, neutral source available to which the disagreeing parties could appeal for settling the target question, only bootstrapping argumentation remains. However, bootstrapping argumentation fails to be persuasive.^37^ Thus, the acquired account of the unpersuasiveness of bootstrapping argumentation also provides an explanation of why certain real-life cases of deep disagreement are irresolvable.^38^

## Possibilities and Limits of Persuasive Argumentation

That bootstrapping argumentation is not a method of rationally persuasive argumentation poses general restrictions for persuasive argumentation for the reliability of a source. Let me next briefly reflect on methods that *are* potentially successful for rationally persuading someone that a particular source *O* is reliable. The persuasiveness of an argumentation that *O* is reliable crucially depends on the fact that B does not doubt that the source to which A refers in arguing is itself reliable. Let me give an example: Suppose that A aims to persuade B via argumentation that thermometer T_1_ is reliable. One potential way of rationally persuading B is by using a second thermometer T_2_ as follows:

**Two thermometers**

(1) A utters (and demonstrates) that T_2_ indicates that the temperature is xº at time t_1_.

(2) B believes that T_2_ is reliable.

(3) B believes that the temperature is xº at t_1_.

(4) A utters (and demonstrates) that T_1_ indicates that the temperature is xº at t_1_.

(5) A utters that, therefore, T_1_ accurately indicates the temperature at t_1_.

(6) B thereby believes that T_1_ accurately indicates the temperature at t_1_.

(7) …

(8) A concludes that T_1_ is reliable. (via induction)

(9) B believes that T_1_ is reliable.

The rational persuasiveness of this argumentation depends on the fact that B believes that the second source, in this case T_2_, is reliable. If B doubts that T_2_ is reliable, and A cannot provide any persuasive supporting argumentation for the reliability of T_2_, then rationally B will not believe information of which B believes that it is delivered by T_2_. Consequently, B will not believe that T_1_ accurately indicates the temperature and, eventually, B will not believe that T_1_ is reliable.

Using a source of the same type, as in the case of two thermometers, is not the only possible way to persuasively argue for the reliability of a source. For example, A can refer to or show B a test report about thermometers of T_1_’s type. If B trusts test reports, then A can thereby argumentatively persuade B that T_1_ is reliable.

Persuasive argumentation for any proposition *p* delivered by *O* faces the same requirement as persuasive argumentation for the claim that* O* is reliable. The two principles RP and RR can explain the following plausible claim about rational persuasion:

Suppose A presents an argument R for *p* to B, consisting of premises *p*_1_…*p*_n_. If B does not already believe *p*_1_…*p*_n_, believes that *p*_1_…*p*_n_ are delivered by a source *O*’ and doubts that *O*’ is reliable, then, in the absence of any supporting arguments, B rationally does not believe that *p* because of R.

This formulation concerns the preconditions and limits of persuasion by using a specific argument (in the absence of specific supporting arguments.) More interestingly, we can also consider the preconditions and limits of persuasive argumentation more generally, i.e., by considering all possible arguments available. There are various ways of persuasively arguing for a proposition *p*. If A presents an argument for *p*, then A can merely utter the premises. In this case, the persuasiveness of A’s argumentation depends on whether B trusts A concerning these premises or whether B already believes them. If not, then A can present evidence for the truth of the premises either by using a source that demonstrates their truth or by mentioning that a source delivers the premises. If A demonstrates the truth of the premises by using a source *O*’ or utters that the premises are delivered by *O*’, then B rationally does not believe these premises on the basis of referring to *O*’ if B doubts that *O*’ is reliable. Hence, persuasive argumentation requires that B trusts A concerning the premises uttered or does not doubt that the sources are reliable to which A refers for supporting the premises (or that B already believes the premises used).

These requirements on persuasive argumentation are plausible and presumably well-known. They jointly explain the following general limitation for persuasive argumentation:

If B does not already believe any of the premises that A can use for arguing for *p*, does not trust A concerning these premises, and doubts the reliability of any source that A can use for delivering premises for an argument for *p*, then A cannot rationally persuade B via argumentation that *p*.

If B does not already believe premises used by A and B’s doubt is holistic in that she suspends judgment about the reliability of any source, including A’s testimony, then there is no way for A to rationally persuade B that *p* and, hence no way to rationally persuade B that* O* is reliable. Put in other words: If B does not already believe the premises, then A can rationally persuade B that *p* only if there is a source that A can use and whose reliability B does not doubt or if B trusts A concerning the premises of A’s argument.^39^ Hence, we can see how the proposed theory on the unpersuasiveness of bootstrapping fits into a larger intuitively appealing picture about the preconditions and limits of rationally persuasive argumentation.

## Conclusion

Bootstrapping is intuitively a flawed reasoning process. One of its defects is that it is not a persuasive way of arguing, i.e., we cannot persuasively argue for the reliability of a source via information from this very source. In this paper, I explain the unpersuasiveness of bootstrapping argumentation by showing that two rules of rationality jointly determine that it is irrational for an interlocutor to believe that a source is reliable on the basis of bootstrapping arguments. The unpersuasiveness of bootstrapping argumentation poses, more generally, restrictions on the possibility of persuasive argumentation.

## Footnotes

1 For an overview, see Weisberg (2012).

2 Vogel presents bootstrapping as a process of inductive reasoning. However, deductive versions of bootstrapping can also be formulated, see Melchior (2016). The following insights about bootstrapping and argumentation are applicable to both inductive and deductive bootstrapping.

3 Bootstrapping is not the only way of Moorean reasoning. Easy knowledge reasoning, as introduced by Cohen (2002 and 2005), can also be regarded as Moorean reasoning. (Actually, Cohen (2002) and (2005) reflects on bootstrapping and easy knowledge reasoning.) In cases of easy knowledge reasoning, three propositions are involved, that *p* seems to be the case, that *p* is true, and that it is not the case that it falsely seems that *p* for some reason *r*. In this paper, I will focus on bootstrapping. For earlier reflections on bootstrapping, see Alston (1986 and 1993), who labelled the problem ‘epistemic circularity’. In this paper, I cannot provide a detailed overview of these discussions and their development.

4 See Pryor (2004 and 2012) and Markie (2005).

5 In this paper, I assume in line with the mainstream of epistemology that argumentation has objective epistemic goals such as knowledge, truth, and/or justification. However, the topic of argumentation theory, which investigates argumentation, is a wide field. It is understood by its proponents to be “an interdisciplinary study in its own right with logical, dialectical, and rhetorical dimensions that are to be nourished by the combined efforts of philosophers, logicians, linguists, discourse analysts, communication scientists, rhetoricians, psychologists, lawyers, and all others who have something to contribute that is theoretically pertinent.” (Van Eemeren et al. 2014, 29) Some branches of argumentation theory significantly overlap with such an epistemological account of argumentation while others substantially diverge. Admittedly, not all areas of argumentation theory are motivated by epistemic questions and not all epistemic questions concern argumentation. Nevertheless, I think that there is a significant overlap between epistemology and argumentation theory and that both disciplines benefit from a mutual exchange. Accordingly, I assume that the results acquired on the basis of epistemological assumptions are of substantial relevance for other approaches in argumentation theory as well. For epistemological reflections on argumentation and epistemological argumentation theories, see Feldman (1994 and 1999), Goldman (1994, 1999), Biro and Siegel (2006), Siegel and Biro (1997 and 2008), and Walton (2013). For an overview of epistemological argumentation theories, see Lumer (2005). For the pragma-dialectical theory of argumentation, which Lumer classifies as a consensus theory of argumentation, see van Eemeren and Grootendorst (1984, 1992 and 2004). For a rhetoric theory of argumentation, see Perelman and Olbrechts-Tyteca (1969). These accounts of argumentation define different goals of argumentation and also provide different definitions of argument and argumentation. For an overview of various argumentation theories, see van Eemeren et al. (2014).

6 In this paper, I focus on verbal persuasion and, accordingly, ignore other forms of argumentation, e.g., visual argumentation. If one takes these forms of argumentation into account, then the provided definition must be understood as one restricted to *verbal persuasion*. For an overview of visual persuasion, see Groarke, Palczewski, and Godden (2016).

7 In this paper, I develop on the working hypothesis that argumentation aims at persuading interlocutors in the sense of changing their mental attitudes concerning *p*. Some authors in argumentation theory are suspicious about these internal goals and prefer a more externalized conception of argumentation and persuasion. Van Eemeren and Grootendorst (2004, 54f), for example, externalize crucial concepts for argumentation such as acceptance and disagreement and understand them as public commitments rather than as mental states. See also Freeman (2005) and Walton (2013). For a critical discussion of this externalized view, see Godden (2010). In epistemology, disagreement and persuasion are usually spelled out in mental, doxastic terms rather than in terms of social commitment, and in this paper, I follow this account. However, nothing substantial hinges on this decision. The central claims of this paper can, mutatis mutandis, also be formulated in terms of external commitments.

8 Issues of causal overdetermination come up concerning persuasion. A’s action *c* might be one cause among others of B’s belief that *p*. B might already believe that *p* and still believe that *p* because of *c*. In this case, *c* is a causal sustainer. Hereinafter, I will exclude such cases of causal overdetermination and assume that A persuades B that *p* via *c* only if B does not believe that *p* prior to being confronted with *c*.

9 One might object that rejecting that *p* is a negative doxastic attitude towards *p* that does not collapse into believing that ~ *p*, for example because S fails to fulfill minimal standards of coherence. I think it is disputable whether such violations of coherence are possible, so for the sake of simplicity I will accept this equivalence.

10 In this paper, I focus on the orthodox doxastic attitudes of believing, rejecting and suspending judgment (plus unawareness), since they fit with our verbal behavior in argumentation situations of affirming or denying a conclusion or of suspending judgment about it. However, convictions come in degrees and one might favor a more fine-grained account of doxastic attitudes. Plausibly, bootstrapping argumentation not only fails to make someone believe that the target source is reliable but also fails to increase the strength of one’s belief (or one’s credence). I assume that we can refine the account presented here such that it also explains this phenomenon.

11 The provided characterization of persuasion understands persuasion as a phenomenon leading to a positive doxastic attitude towards *p*. Changes of doxastic attitudes are not only possible in a positive direction but also in a negative one, i.e. from believing that *p* to suspending judgment or rejecting that *p* or from suspending judgment to rejecting that *p*. I assume for the sake of simplicity that changes of doxastic attitudes in a negative direction relative to *p* are changes in a positive direction relative to ~ *p*. Instances of negative persuasion about the falsity of *p* leading to rejection of *p* are therefore reducible to positive persuasion about the truth of ~ *p*. If one does not accept this reduction, then the following claims about positive persuasion can be formulated mutatis mutandis for negative persuasion.

12 Plausibly two persons do not disagree about *p* if A has a doxastic attitude towards *p* but B is not aware of *p*. Moreover, we can also talk about disagreement if two persons have different credences concerning a proposition but both credences amount to believing that *p* (or both credences amount to rejecting that *p*.) However, these subtleties are not important for the purposes of this paper.

13 For a theory of defeasibility, see Klein (1981). For a coherentist version, see Lehrer (1990).

14 I use here the notion of persuasion in a rather loose sense that includes cases of persuasion merely on the basis of trust. Most notions of persuasion are more robust and can involve forms of trust but do not exclusively rely on it. A does not necessarily have to *assert* that *p* in order to persuade B that *p*. Under certain conditions, A can also make B believe that *p* without having any particular intentions. Therefore, I will talk hereinafter about utterances rather than about assertions. Moreover, I only present here a definition of belief forming processes via utterances. I do not defend a theory about the justificatory status of these beliefs. For discussions of these issues, see Lackey (2008), Faulkner (2011), and Kallestrup (2020).

15 This definition of trust might diverge in some cases from a more demanding understanding that regards trust as a reflective capacity. For example, children who do not have a concept of trust but rather automatically believe utterances of their parents might not be properly regarded as trusting their parents according to this understanding.

16 For the purposes of this paper, I need not provide a general account of which arguments are valid or cogent. For a more detailed definition of argumentation, see for example van Eemeren and Grootendorst (2004, 1) who define argumentation as a “verbal, social, and rational activity aimed at convincing a reasonable critic of the acceptability of a standpoint by putting forward a constellation of propositions justifying or refuting the proposition expressed in the standpoint.” See also Walton (2006, 1) who defines argumentation as “the giving of reasons to support or criticize a claim that is questionable or open to doubt.”.

17 Arguments consist of one or more premises and a conclusion. In paradigmatic cases of argumentation, A utters the premises and the conclusion. However, in many everyday cases of argumentation, some premises are tacitly assumed or the conclusion is implicitly suggested. The central claims of this paper are independent of whether all premises and/or the conclusion are explicitly stated.

18 Persuasion on the basis of trust and persuasion via argumentation are two important forms of making someone believe and do not exclude each other. Typical everyday cases of persuasion will involve a combination of both factors.

19 This notion of persuasion via argumentation in closely related to a view on *rational persuasion* defended in the literature. According to this view, a hearer is rationally persuaded by an argumentation to believe that *p* if the argumentation provides reasons to believe that *p* according to the hearer’s own standards of rationality. Reasonably (and presumably also usually), subjects behave such that they believe the conclusion of an argumentation because of the argumentation iff they believe that the argumentation provides reasons to believe the conclusion. Otherwise, the subjects would form beliefs against their own standards of rationality. In this sense, there is a close connection between persuasion via argumentation as defined here and the concept of rational persuasion. For different versions of this view of rational persuasion, see Vorobej (2006), Lynch (2010, 2016), Ranalli (2020) and Simard Smith and Michael (2020). For a more detailed investigation of interlocutors who follow their own standards of rationality, see Melchior (2023a and 2023b), where I call  them subjectively rational.

20 We can distinguish a narrow scope reading of rationality requirements from a wide scope reading. On the narrow scope reading, for example, given that S believes that *p* and believes that *p* entails *q*, it is rational for S to believe that *q*. On a wide scope reading, it is irrational to believe that *p* and believe that *p* entails *q* without believing that *q*. Nothing here hinges on whether we opt for a narrow scope reading or a wide scope reading. For a discussion, see Kolodny (2005) and Broome (2005).

21 For a similar conception of rationality, see Pryor (2004). In order to defend Mooreanism, Pryor distinguishes rationality requirements as defined here from principles of justification transmission. He suggests that reasoning processes such as Moorean reasoning violate principles of rationality nevertheless they can transmit justification. See also Pryor (2012). However, Pryor reflects on what is rational for S to believe given actual entailment relations whereas I focus on the purely subjective level and on entailment relation that the subject *believes* to hold.

22 B might believe some but not all premises of a presented argument and still believe that *c* because of the argument if B thinks that these premises already offer sufficient support for *c*. In order to cover these cases, RP had to be reformulated as follows: RP’ – B usually believes that *c* because of A’s argumentation consisting of premises *p*_1_…*p*_n_ and conclusion *c*, only if B believes *some* of the premises *p*_1_…*p*_n_. For the purposes of this paper, version RP is sufficient.

23 A might also fail to rationally persuade B that *p* via argumentation R if B does not believe that the premises of R sufficiently support the conclusion that *p*. Hence, agreement about validity or cogency of arguments is an essential requirement for persuasive argumentation. This agreement need not be explicitly conceptualized in terms of validity or cogency. Rather, A and B might simply agree that R is a good argument for *p*. For an investigation of this requirement for persuasive argumentation, see Melchior (2023a). I assume hereinafter that A and B at least implicitly agree about the quality of the arguments presented. For similar reflections on conceptual requirements, see Bergmann (2006, 162.).

24 For a similar intuition in the context of settling a question, see Davies (2004).

25 If we suspend judgment about whether *i* is delivered by *O*, then the situation is different. In this case, we presumably also suspend judgement about the truth of *i* if we do not believe that* O* is reliable.

26 There are two different takes on the nature of defeaters. On the one hand, for epistemologists such as Klein (1981) who argue that knowledge is undefeated true belief, defeaters are true propositions. On the other hand, those who think that defeaters concern one’s justification regard defeaters as mental states. For the purposes here, the analogy to mental state defeaters, not to propositional defeaters, is crucial.

27 It is disputable whether such an undercutting mental state defeater also removes one’s *justification* to believe that *p*. Suppose that S believes that *p* on the basis of *e* and acquires the belief that *e* does not provide evidence for *p*. Does this belief remove S’s justification to believe that *p* even if the belief in the undercutting defeater is entirely unjustified? Presumably, many will answer this question in the negative, for example Pryor (2004). Bergmann (2006), however, defends this view. I am here only committed to the weaker claim that it is *irrational* for S to believe that *p* on the basis of *e* if S believes an undercutting defeater.

28 In the second case, however, the bootstrapping nature of A’s argumentation would become transparent to B.

29 Moreover, A could also support her claim for (1) by pointing at the indication of the gas gauge, which would not be a process of pure argumentation anymore.

30 One might suggest that Moorean reasoning need not be reconstructed as a dialogue with an inner skeptic since a real person can also doubt the reliability of someone else’s sense apparatus. However, reconstructions of Moorean reasoning as argumentations between two real persons A and B are problematic. In particular, if B doubts the reliability of her whole sense apparatus then she will doubt the existence of A and *for this reason* B will trivially fail to believe A’s argumentation rather than because she does not regard A’s argumentation as persuasive. For the concept of inner dialogues between a believer and a skeptic, see Dutilh Novaes (2016).

31 This paper is not the place for an elaborated investigation of such cases. For the view, that the irresolvability of deep disagreement is based on the dialectical defectiveness of bootstrapping, see Lynch (2010 and 2016).

32 Perner (1991) argues that children acquire the concepts of representation, which are necessary for acquiring concepts of reliability of information delivering sources, at the age of around four years.

33 As already mentioned, it is not crucial for the purposes of this paper to draw a sharp distinction between unawareness and implicit doxastic attitudes.

34 It is a different question whether S can *justifiedly* believe information if S does not believe that the information delivering source is reliable. I think it is highly plausible to ascribe justification and knowledge to children who do not have concepts about the sources that deliver the information which the children believe. Thus, I think that the claim reformulated about what subjects justifiedly believe instead of about what subjects rationally believe is also false. Nothing for the purposes of this paper, however, hinges on this issue.

35 Actually, everybody (or at least almost everybody) who possesses the concept of a sense apparatus also believes that her own sense apparatus functions reliably (at least most of the time). These beliefs are plausibly not innate but acquired. Moreover, they are plausibly not formed via a priori reasoning. Hence, they are acquired via a form of Moorean reasoning, presumably in a complex way that also relies on background assumptions. Hence, there *is* a persuasive instance of Moorean *reasoning*. However, there is a difference between arguing and reasoning. While a specific type of Moorean reasoning can be persuasive, Moorean *argumentation* understood as a process that mimics actual argumentation between two interlocutors is not. For a more detailed analysis, see Melchior (2020). These issues about the persuasiveness of Moorean reasoning and Moorean argumentation must be distinguished from issues concerning the transmission of justification via Moorean reasoning. I do not commit myself here to any claim about whether justification transmits via bootstrapping.

36 The notion of deep disagreement was introduced by Fogelin (1985) in his pioneering work. For an overview of the discussion, see Ranalli and Thirza (2022a and 2022b). For the connection between deep disagreement and bootstrapping argumentation, see Lynch (2010 and 2016) and Melchior (2023a and 2023b).

37 Using the conceptual tools of argumentation theory, we can describe the argumentative fallacy as coming close to or being related to the fallacy of begging the question in the sense of only accepting one’s own sources or to argumentum ad verecundiam of only accepting one’s own authorities. Due to lack of space, these connections cannot be investigated here in detail.

38 Further real-life cases of deep disagreement can involve disagreement between a conspiracy theorist and a non-conspiracy theorist about the reliability of “the media” or of conspiracy bubbles in the internet. Note that these real-life cases of disagreement are not instances of peer disagreement, i.e., instances of disagreement between two parties who are epistemic peers or at least consider each other as such. In cases of peer disagreement, the parties tend to doubt their own views in light of disagreement with the epistemic peer. (Whether they rationally do so, is subject of discussion.) Such mechanisms do not apply to the real-life cases discussed here.

39 For an analysis of the limits of argumentation in the context of deep disagreement, see Melchior (2023a and 2023b).
